# Age-appropriate potassium clearance from perinatal cerebrospinal fluid depends on choroid plexus NKCC1

**DOI:** 10.1186/s12987-023-00438-z

**Published:** 2023-06-16

**Authors:** Ryann M. Fame, Huixin Xu, Aja Pragana, Maria Lehtinen

**Affiliations:** 1 Department of Pathology, Boston Children’s Hospital, Harvard Medical School, Boston, MA 02115 USA; 2 Present Address: Department of Neurosurgery, Stanford University, Stanford, CA 94305 USA

**Keywords:** Choroid plexus, Cerebrospinal fluid, Potassium clearance, NKCC1, Genome editing, AAV, Conditional knockout, Brain development, Cerebral cortex

## Abstract

Regulation of the volume and electrolyte composition of the cerebrospinal fluid (CSF) is vital for brain development and function. The Na-K-Cl co-transporter NKCC1 in the choroid plexus (ChP) plays key roles in regulating CSF volume by co-transporting ions and mediating same-direction water movements. Our previous study showed ChP NKCC1 is highly phosphorylated in neonatal mice as the CSF K^+^ level drastically decreases and that overexpression of NKCC1 in the ChP accelerates CSF K^+^ clearance and reduces ventricle size [1]. These data suggest that NKCC1 mediates CSF K^+^ clearance following birth in mice. In this current study, we used CRISPR technology to create a conditional NKCC1 knockout mouse line and evaluated CSF K^+^ by Inductively Coupled Plasma Optical Emission spectroscopy (ICP-OES). We demonstrated ChP-specific reduction of total and phosphorylated NKCC1 in neonatal mice following embryonic intraventricular delivery of Cre recombinase using AAV2/5. ChP-NKCC1 knockdown was accompanied by a delayed perinatal clearance of CSF K^+^. No gross morphological disruptions were observed in the cerebral cortex. We extended our previous results by showing embryonic and perinatal rats shared key characteristics with mice, including decreased ChP NKCC1 expression level, increased ChP NKCC1 phosphorylation state, and increased CSF K^+^ levels compared to adult. Collectively, these follow up data support ChP NKCC1’s role in age-appropriate CSF K^+^ clearance during neonatal development.

## Background

Abnormal CSF accumulation, as occurs in hydrocephalus, can negatively affect perinatal neurodevelopment. However, we lack a clear understanding of the mechanisms involved in regulating CSF secretion and clearance during the critical postnatal period prior to the formation of lymphatic systems. Previous work has demonstrated that NKCC1, a bi-directional Na^+^-K^+^-Cl^−^ co-transporter expressed on the apical surface of choroid plexus (ChP) epithelial cells, contributes to adult cerebrospinal fluid (CSF) production [2–11]. In contrast, our recent work in developing mice demonstrated that ChP-NKCC1 reduces CSF volume during perinatal development [1]. The observed shift in ChP NKCC1-dependant net ion transport direction can be reconciled by two considerations. First, the direction of NKCC1 transport is determined by the combined electrochemical gradients of Na^+^, K^+^, and Cl^−^. Second, CSF ionic composition can vary under different circumstances. Given that embryonic mouse has a relatively high CSF K^+^ concentration and that developing ChP expresses high levels of the active phosphorylated form of NKCC1, p-NKCC1, in the perinatal time period when CSF K^+^ decreases to adult levels [1], we hypothesized that ChP-NKCC1 was mediating CSF ion absorption. We tested this idea with a gain-of-function approach using a hybrid AAV serotype with tropism for ChP epithelial cells (AAV2/5) to overexpress NKCC1 [1, 12]. We found that increased ChP NKCC1 reduces both the CSF volume and K^+^ concentration during normal development, and further mitigates CSF volume increases in a mouse model of obstructive hydrocephalus [1] and of post-hemorrhagic ventriculomegaly [13]. Although suggestive, our data did not directly demonstrate that endogenous ChP NKCC1 is necessary for normal development or adaptive responses to excessive CSF accumulation. To address these questions, we developed methods to achieve specific inhibition of ChP NKCC1 in mice by embryonic knockdown. We also recapitulated in rats our previous observations regarding CSF ion concentrations during mouse development, suggesting that the mechanisms are conserved between species.

## Methods

### Animals

The Boston Children’s Hospital IACUC approved all experiments involving mice and rats in this study. Mice with germline loxP-*Slc12a2*-loxP were generated in-house by Boston Children’s Hospital Mouse Genetic Manipulation Core on C57BL6 background. LoxP sites were inserted to flank exons 5 and 6. When exons 5 and 6 are removed by Cre-recombinase, a frame shift occurs and leads to an early stop codon in exon 7. To insert LoxP sites, a single-stranded DNA homology-directed repair (HDR) template was designed to span exons 5 and 6 with one LoxP site on each end. Guide RNAs (gRNA) were designed to target the sequences at both 5’ and 3’ ends of the HDR template and checked to ensure they are unique in the mouse genome. The template (produced by Genescript), gRNA (by IDT), and CRISPR-Cas9 nuclease (IDT #1,081,060) were injected into mouse zygotes. Mice harboring correct LoxP insertions were screened by genotyping PCR with a primer pair targeting the LoxP sequence (no bands in wild type [WT] mice) and a primer pair flanking the correct LoxP insertion sites (smaller bands in WT mice). The founder mouse was sequenced to confirm the absence of unintended genetic alterations beyond the LoxP insertion sites within the HDR targeted region. The founder mouse was bred to WT C57BL6 for 5 generations (F1-F5). Progenies from F6 and later were used for experiments. Genotyping primers are: AGTCCTGTGGATTCTGCCCT (Slc12a2-5P-F); GCATCACTCACTCCCATGCATAAC (Slc12a2-5PloxP-R); ACATGAGACCAAGCAGCACA (Slc12a2-5P-R); TGAGCCGTGCTCTAGGAAAC (Slc12a2-3P-F); GCCCAAGGCCCCTTGATAACT (Slc12a2-3PloxP-F); AAGCCCTAAAGACGGGCAAA (Slc12a2-3P-R).

Sprague Dawley rats were purchased from Charles River Laboratories. Animals were housed in a temperature-controlled room on a 12-hr light/12-hr dark cycle and had free access to food and water. In cases where the studies involved mice and rats younger than postnatal day (P) 10, all animals were allocated into groups based solely on the gestational age without respect to sex (both males and females were included). For older animals, equal number of males and females were included.

### *In utero* intracerebroventricular (ICV) injection

Timed pregnant mice (E14.5) were anesthetized by isoflurane and placed on warm pads. Laparotomy was performed and the uterine horns were exposed. Virus solution (1 µl, with a titer of 1 × 10^12^) was injected into one lateral ventricle of each embryo using glass capillary pipettes [14]. Immediately following injection, the uterine horns were placed back into the abdominal cavity and the incision was sutured.

### AAV production

AAV-Cre was purchased from BCH viral core at Boston Children’s Hospital. AAV-Null was purchased from Vector Biolabs (Pennsylvania, USA).

### Tissue processing

Samples were fixed in 4% paraformaldehyde (PFA). For cryosectioning, samples were incubated in the following series of solutions: 10% sucrose, 20% sucrose, 30% sucrose, 1:1 mixture of 30% sucrose and OCT (overnight), and OCT (1 h). Samples were frozen in OCT.

### Immunostaining

Cryosections were blocked and permeabilized (0.3% Triton-X-100 in TBS; 5% serum), incubated in primary antibodies overnight and secondary antibodies for 2 h. Sections were counterstained with Hoechst 33,342 (Invitrogen H3570, 1:10000) and mounted using Fluoromount-G (SouthernBiotech). The following primary antibodies were used: CTIP2 (abcam, ab54583, 1:100); TBR1 (abcam, ab31940, 1:200); Satb2 (abcam, ab51502, 1:100); Olig2(R&D Systems, AF2418, 1:100): S100b (Dako, Z0311, 1:200). Antigen retrieval in hot citric acid (pH6.0) was used for CTIP2 and TBR1. Secondary antibodies were selected from the Alexa series (Invitrogen, 1:500). Images were acquired using Zeiss LSM880 confocal microscope with 20 x objective.

### Immunoblotting

Tissues were homogenized in RIPA buffer supplemented with protease and phosphatase inhibitors. Protein concentration was determined by BCA assay (Thermo Scientific 23,227). Samples were denatured in 2% SDS with 2-mercaptoethanol by heating at 37 °C for 5 min. Equal amounts of proteins were loaded and separated by electrophoresis in a 4–15% gradient polyacrylamide gel (BioRad #1,653,320) or NuPAGE 4–12% Bis-Tris gel (Invitrogen #NP0322), transferred to a nitrocellulose membrane (250 mA, 1.5 h, on ice), blocked in filtered 5% BSA or milk in TBST, incubated with primary antibodies overnight at 4 °C followed by HRP conjugated secondary antibodies (1:5000) for 1 h, and visualized with ECL substrate. For phosphorylated protein analysis, the phospho-proteins were probed first, and then blots were stripped (Thermo Scientific 21,059) and reprobed for total proteins. The following primary antibodies were used: rabbit anti-NKCC1 (Abcam ab59791; 1:1000), rabbit anti-pNKCC1 (EMD Millipore ABS1004; 1:1000), rabbit anti-GAPDH (Sigma G9545; 1:10000).

### Quantitative RT-PCR

For mRNA expression analyses, the ChP or cortical hemispheres were dissected from individual pups. RNA was isolated using the RecoverAll Total Nucleic Acid Isolation Kit (Life Technologies AM1975) for ChP following manufacturer’s specifications, omitting the initial FFPE-specific treatments. Extracted RNA was quantified spectrophotometrically and 100 ng was reverse-transcribed into cDNA using the High Capacity cDNA Reverse Transcription kit (Applied Biosystems #4,368,814) following manufacturer’s specifications. RT-qPCRs were performed in duplicate using Taqman Gene Expression Assays and Taqman Gene Expression Master Mix (Applied Biosystems) with Rplp0 as an internal control. Cycling was executed using the StepOnePlus Real-Time PCR System (Invitrogen) and analysis of relative gene expression was performed using the 2^−ΔΔCT^ method. Technical replicates were averaged for their cycling thresholds and further calculations were performed with those means.

### Fluid collection and potassium detection

CSF was collected by a glass capillary inserted into the cisterna magna. In brief, viable embryos were collected rapidly after their removal from the dam (within 15 min), with placenta attached. Neonatal rats were anesthetized either by hypothermia (P0-P1) with CSF collected within 2–3 min (the timeframe when anesthetized pups can recover with < 30 s of warming) or ketamine/xylazine (P4 and above). CSF was centrifuged at 1000 x g for 10 min at 4 °C to remove any tissue debris. Pellets were inspected to ensure lack of blood cells. Potassium quantification was performed by Galbraith Laboratories, Inc (Knoxville, TN, USA), using inductively coupled plasma - optical emission spectrometry (ICP-OES). All tests were performed using 5–7µL of CSF. CSF was pooled from younger animals within a single litter to enable metals quantification. Blood was collected by cardiac puncture. Blood was allowed to coagulate at room temperature and centrifuged at 1000 x g for 10 min at room temperature, supernatant (serum) was collected and used for ICP-OES. To test if hypoxia in embryos alters CSF and serum measurements, addition samples were collected with the embryos attached to dams under anesthesia by ketamine/xylazine.

### Cortical cell quantification

Sections from the cerebral cortex were imaged with a 5 x objective on an inverted fluorescent light microscope (Zeiss). A rectangle was divided into 10 equally sized bins and placed over the somatosensory cortex. The box height was scaled so the top (bin 10) was tangential to the top of layer I and the bottom (bin 1) was tangential to the bottom of layer VI. Positive cells were counted in each bin by a blinded researcher as described in [15]. Cell numbers in each bin were scaled by the scaling factor of the box to account for slight differences in overall cortical thickness. For cortical markers, numbers are reported as percentages of the total number of counted cells.

### Quantification and statistical analyses

Biological replicates (N) were defined as samples from distinct individual animals, analyzed either in the same experiment or within multiple experiments, except when individual animals could not provide a sufficient sample (i.e., CSF), in those cases multiple animals were pooled into one biological replicate and the details are stated in the corresponding figure legends. Statistical analyses were performed using Prism 7. Outliers were excluded using ROUT method (Q = 1%). Appropriate statistical tests were selected based on the distribution of data, homogeneity of variances, and sample size. The majority of the analyses were done using Welch’s unpaired t-test, with correction for multiple comparison. Data are presented as means ± standard deviation (SD). Please refer to figure legends for sample size. *p* values < 0.05 were considered significant (* *p* < 0.05, ** *p* < 0.01, *** *p* < 0.001, **** *p* < 0.0001). Exact p values can be found in the figure legends. P values are also marked in the figures where space allows.

## Results

### Creation of flox-Slc12a2 mice and conditional ChP NKCC1 knockdown with AAV-Cre

**Fig. 1:**
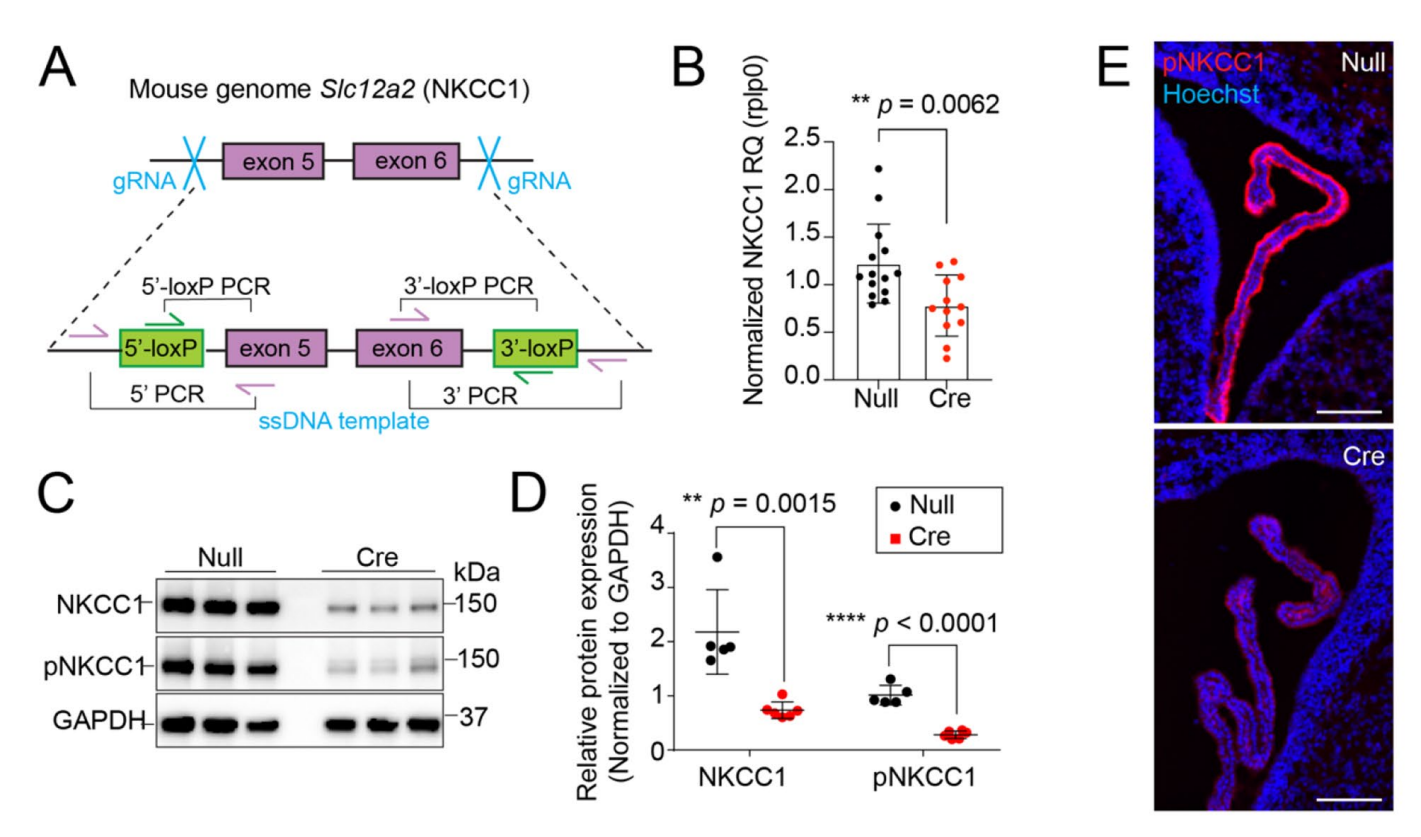
Creation of NKCC1 conditional knockout mouse line (**A**) Schematics of CRISPR design and genotyping strategy. LoxP sites were inserted to flank exon 5 and exon 6 of *Slc12a2* (NKCC1) gene. (**B**) qPCR showing reduced NKCC1 transcripts in neonatal mice following embryonic ChP NKCC1 knockdown by AAV-Cre vs. AAV-Null (empty). N = 13 AAV-Null and N = 11 AAV-Cre. ** *p* = 0.0062. (**C**) Immunoblotting of P4 ChP showing reduction in total and pNKCC1 by AAV-Cre. (**D**) Quantification of immunoblots. N = 4 for each condition. ** *p* = 0.015, **** *p* < 0.0001. Welch’s t-test. (**E**) Representative images showing robust pNKCC1 in AAV-Null mice but not in AAV-Cre mice. Scale = 100 μm

To investigate whether ChP NKCC1 was necessary for CSF K^+^ clearance, we created a conditional NKCC1 knockout strain using genome editing [16] where LoxP sites were inserted flanking exons 5 and 6 of the *Slc12a2* gene (Fig. 1A). Founders were positively screened for correct LoxP insertion and negatively screened for undesirable mutations outside of the LoxP sequences. The founder mouse was bred to wild-type C57/BL6 for 5 generations prior to experiments. To trigger Cre-mediated recombination and NKCC1 deletion in developing ChP epithelial cells, we performed in utero (ICV) injection of AAV-Cre at embryonic day 14.5 (E14.5). We used AAV2/5 with demonstrated tropism for ChP epithelial cells [1, 12]. AAV-Null containing the pre-packaged AAV, CAG promoter, but no transgene was used as a negative control. ChP was harvested from these mice at P4 to determine efficacy of knockout. AAV-Cre resulted in decreased ChP-NKCC1 expression at the transcript and protein levels (Fig. 1B-D) leading to an overall knockdown of NKCC1 in the P4 ChP. Immunostaining confirmed that mice injected with AAV-Cre had very low levels of pNKCC1 in their ChP compared to controls (Fig. 1E).

### Mice with embryonic ChP-targeted NKCC1 knockdown showed delayed CSF K^+^ clearance

**Fig. 2:**
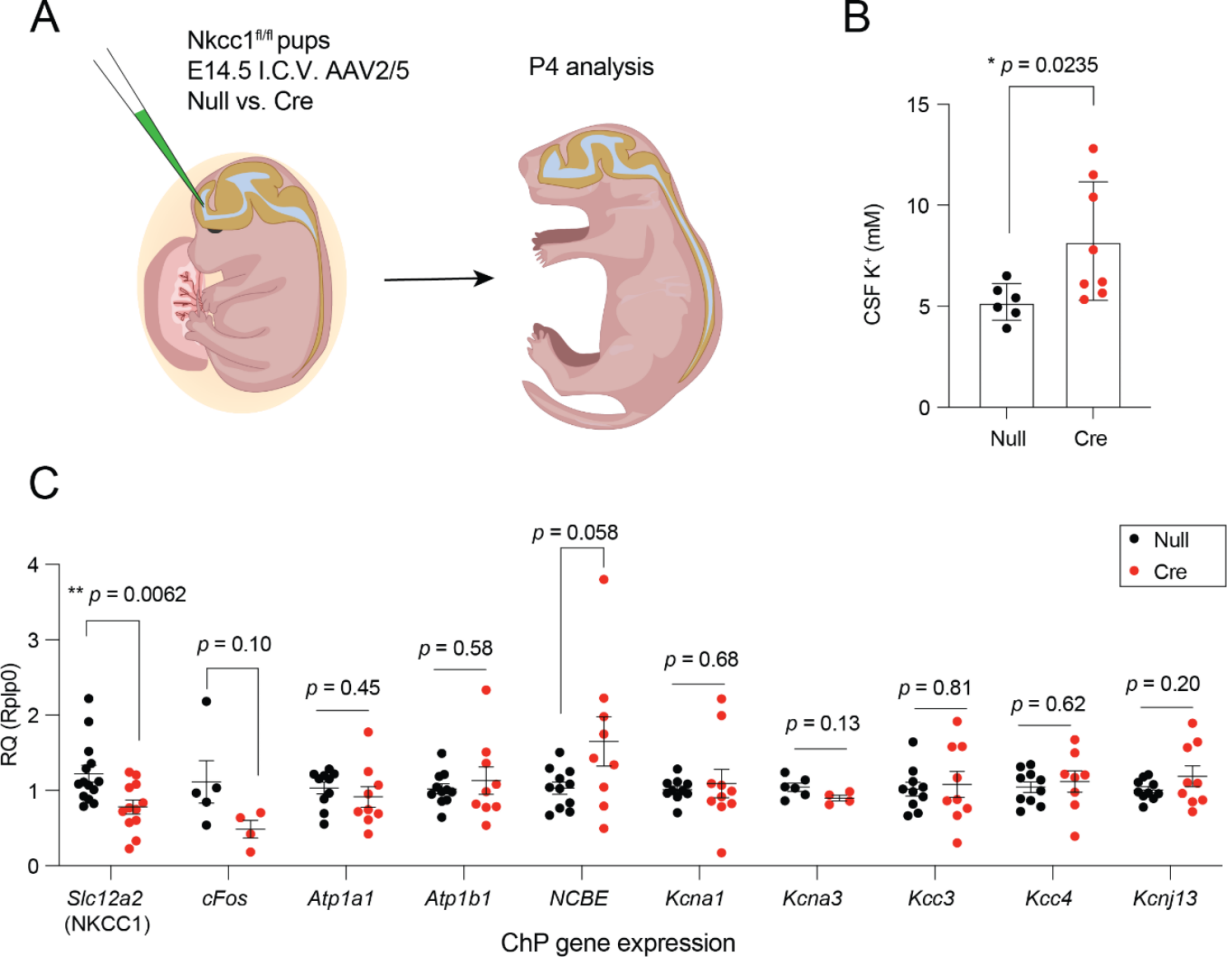
NKCC1 conditional knockdown in ChP delays perinatal CSF potassium clearance (**A**) Schematic of ICV injection of AAV2/5 AAV-Cre vs. AAV-Null (empty) viral vectors at E14.5 followed by analysis of CSF, ChP, and cortex at P4. (**B**) CSF [K^+^] was significantly elevated at P4 from mice with NKCC1 knockdown as determined by ICP-OES. N = 6 for AAV-empty; N = 8 for AAV-Cre. (**C**) No other transporters or channels that modulate K^+^ were changed after NCKK1 knockdown. * *p* < 0.05, ** *p* < 0.01. Welch’s t-test

Mice with embryonic NKCC1 knockdown beginning at E14.5 had higher CSF [K^+^] levels at P4 compared to controls (Fig. 2A,B). These levels were similar to P0 CSF [K^+^] values that we published previously and much higher than typical P4 levels, which are closer to adult values [1]. We did not observe changes in mRNA levels for other K^+^ channels or transporters (Fig. 2C), suggesting no compensatory effects from overexpressing other channels due to any developmental stress, although we cannot rule out the possibility that protein levels or activation status of these other transporters and channels could still be different. We attribute the variability in CSF K^+^ values to the partial knockdown that is achieved at this age by the AAV-Cre delivery approach (Fig. 1C, D). These data are consistent with NKCC1 being necessary for the observed perinatal decrease in CSF [K^+^] and complement our previous findings that embryonic NKCC1 overexpression induces a premature reduction in CSF [K^+^] [1].

### Mice with embryonic ChP-targeted NKCC1 knockdown had grossly normal cerebral cortices

**Fig. 3:**
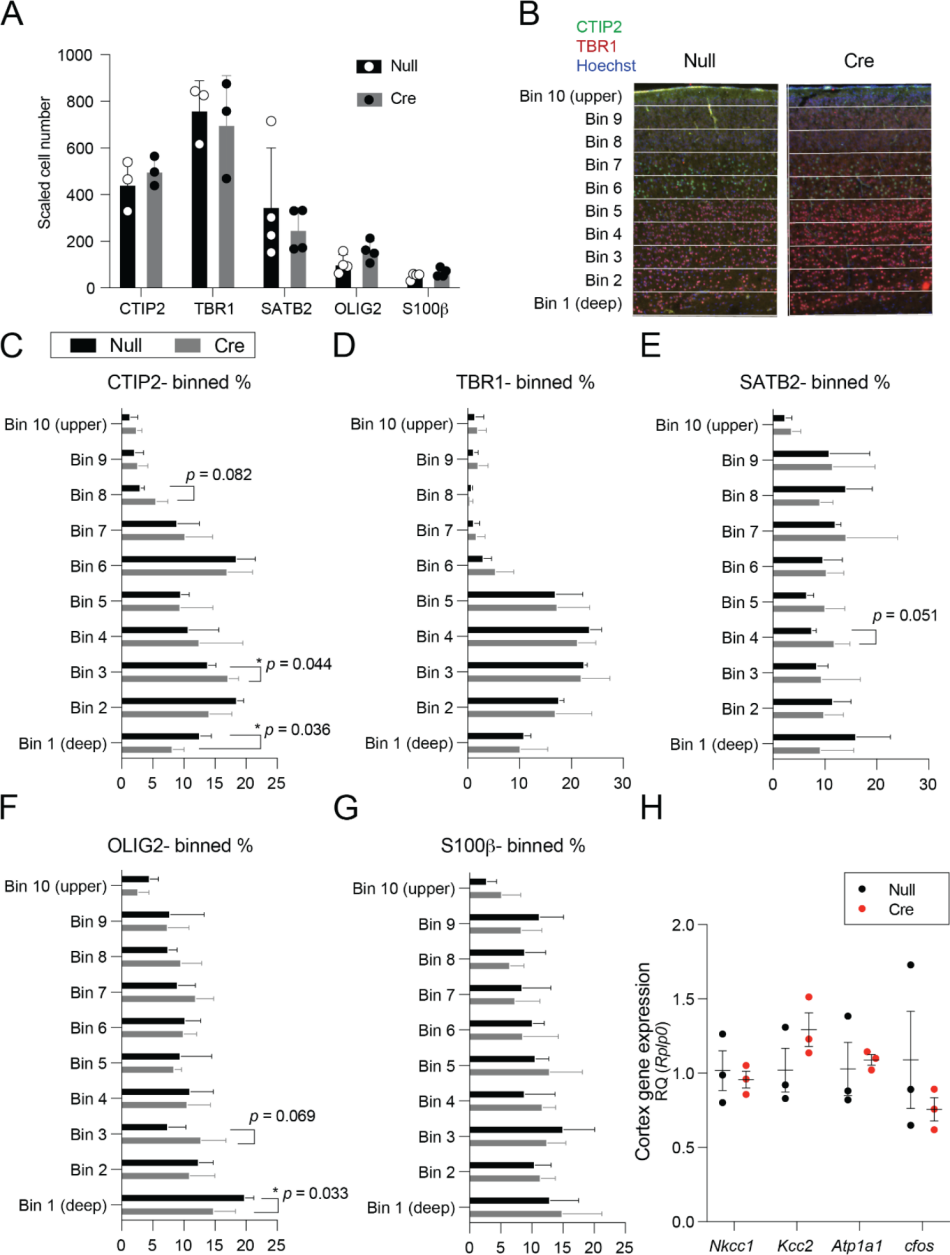
Effects of NKCC1 conditional knockdown in ChP on cortical development. (**A**) Total counts of TBR1 TBR2 SATB2 OLIG2 and S100β expressing cells in S1 neocortex at P4 scaled for cortical thickness. (**B**) Example of the 10-bins used for subsequent quantification. (**C-G**) Binned shifts in cortical cell types. N = 3–4 per condition. (**H**) No observed cortical gene expression changes after ChP NKCC1 knockdown. N = 3 per condition. * *p* < 0.05, ** *p* < 0.01. Welch’s t-test

We did not observe any overall difference in cerebral cortical thickness or in cortical projection neuron cell type abundance in ChP-NKCC1 knockdown brains compared to controls. Analyses included layer VI corticothalamic neurons (Tbr1^+^), layer V and VI corticofugal neurons (Ctip2^+^), and non-corticofugal cortical neurons (Satb2^+^) (Fig. 3**A**). Neither did we observe any differences in the later born glial cell types including oligodendroglia (Olig2^+^) and astroglia (S100β^+^) nor the overall number of cortical glia that developed after ChP-NKCC1 knockdown was induced (Fig. 3A). We also binned the cortex into 10 equidistant bins (Fig. 3B) to investigate cell-type specific migration. The distribution of cellular identities across cortical layers at P4 did not largely change (Fig. 3C-G), however deep layer distribution of Citp2^+^ and Olig2^+^ cells did exhibit slight shifts (Fig. 3C & F). *Slc12a2* (NKCC1), *Slc12a5* (KCC2), *Atp1a1* (Na^+^ K^+^-Atpase) and *c-fos* expression were unchanged in bulk cortex (Fig. 3H). While NKCC1 mutations are associated with severe neurodevelopmental disorder [17], our data suggest that neither the ChP-targeted NKCC1 knockdown nor this prolonged timeframe of high CSF [K^+^] grossly influenced neocortical lamination or cell-type generation by P4.

### Patterns of CSF and serum K^+^ clearance in developing rats were like those in developing mice

**Fig. 4:**
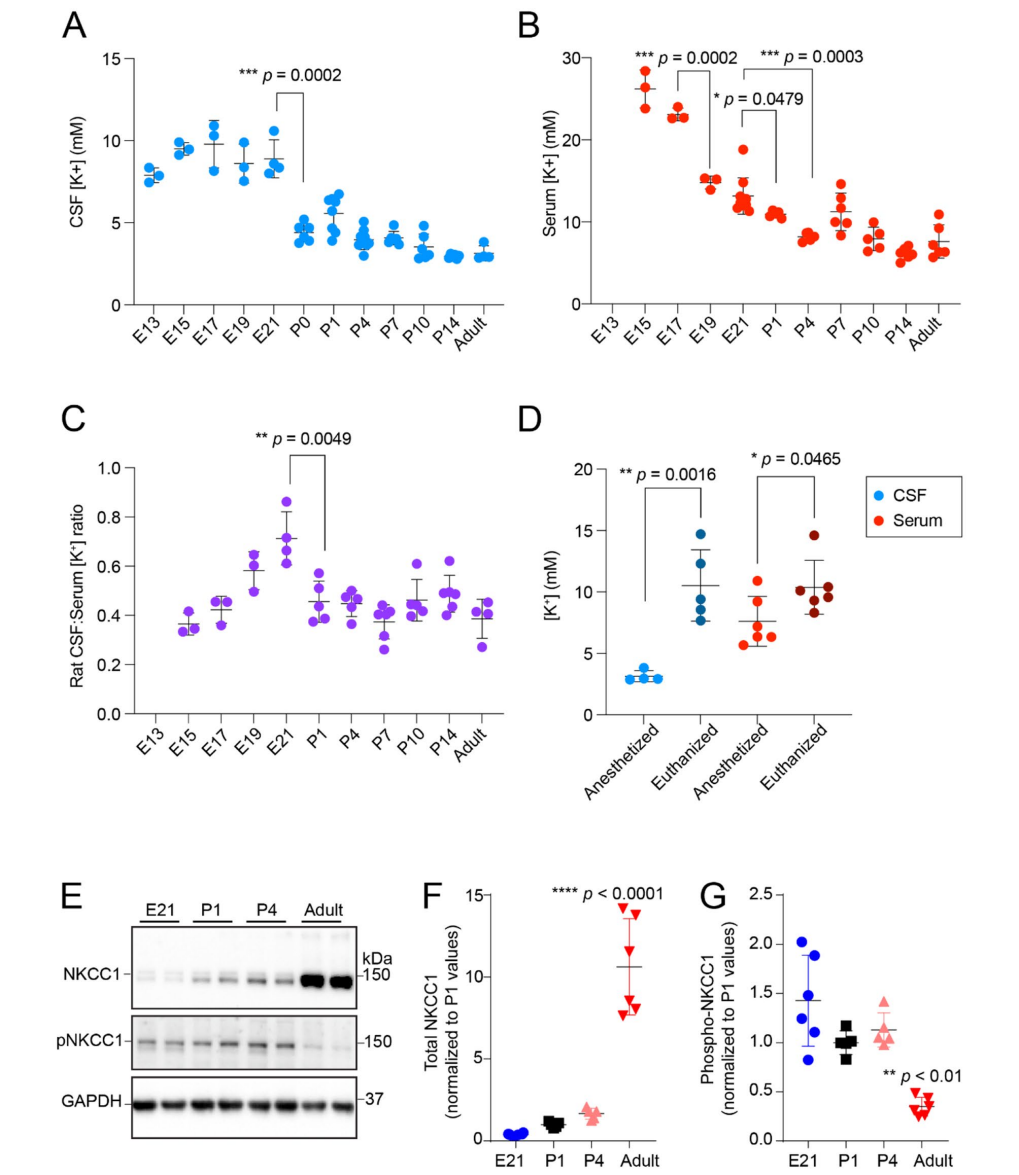
Studies in rats recapitulated CSF K^+^ and ChP NKCC1 findings in mice (**A**) Developing rat CSF had significantly decreased K^+^ concentration from E21 to P1. N = 3–7. **** *p* = 0.0002; (**B**) Developing rat serum had significantly decreased K^+^ concentration from E17 to E19, and then to P4. *** *p* = 0.0.0002 (E17 to E19); * *p* = 0.0479 (E21 to P1); *** *p* = 0.0003 (E21 to P4). (**C**) CSF:serum K^+^ ratio showed beginning of CSF clearance around P1. N = 4–6. *** *p* = 0.0049. (**D**) CSF and serum collected from euthanized adult rats had significantly higher K^+^ concentration than those collected from anesthetized live adults. *** *p* = 0.0016; * *p* = 0.0465. Statistics in A-D were calculated by unpaired t-test with Welch’s correction. (**E-G**) Rat ChP immunoblotting showing increased total NKCC1 and decreased pNKCC1 during development. N = 5–6 per condition. F-G show quantification of all samples. F: **** *p* < 0.0001 (one-way ANOVA with multiple comparison. E21 vs. adults: P1 vs. adults: ; P4 vs. adults). G: ** *p* < 0.01 (one-way ANOVA with multiple comparison. E21 vs. adults: <0.0001; P1 vs. adults: 0.0042; P4 vs. adults: 0.0007)

Our finding in mice that activation of NKCC1 elicits robust decreases in CSF K^+^ over a narrow perinatal time window may have translational implications if it is conserved across species. We therefore repeated our measurements in rats with high temporal resolution during embryonic and neonatal development. We sampled serum and CSF from developing rats at E13, E15, E17, E19, E21, P0 or P1, P4, P7, P10, P14, and adult. Both serum and CSF were collected for ICP-OES analysis to quantify the K^+^ concentration. We found the same temporal decrease of CSF K^+^ concentration from E21 to P1 in perinatal rats (Fig. 4A), in concert with a decrease in serum K^+^ concentration (Fig. 4B), and the CSF to serum K^+^ ratio (Fig. 4C), suggesting the onset of CSF clearance.

Extracellular K^+^ levels increase following hypoxia [18]. Consistent with this understanding, we observed that CSF collected from CO_2_ euthanized adults had an abnormally high K^+^ concentration compared to CSF collected from live adults under anesthesia (Fig. 4D). To determine if embryonic CSF displayed similar levels of sensitivity to CO_2_ euthanasia as dams, we compared CSF samples collected from embryonic mice from CO_2_ euthanized vs. ketamine/xylazine anesthetized dams (in both cases, the embryos were confirmed as viable [heartbeat at sample collection]). Importantly, these maternal states did not influence embryonic CSF K^+^ at E14.5 (7.42 ± 1.17 mM from euthanized dam vs. 6.05 ± 1.29 mM from anesthetized dam, multiple samples were pooled from over 10 embryos under each condition, *p* = 0.34). In contrast, maternal state did influence embryonic serum K^+^, and at E17.5, serum K^+^ increased in embryos of euthanized dams (18.04 ± 1.75 mM from euthanized dam vs. 12.77 ± 1.90 mM from anesthetized dam, *p = 0.02; no observed trends of K^+^ increase from first sample to last). Adult serum K^+^ showed the highest sensitivity to CO_2_ euthanasia, rising to 19.46 ± 4.02 mM, consistent with prior reports [18]. Collectively, these values demonstrate that while sample collection methods should be carefully considered when interpreting data, embryonic CSF K^+^ collected by the approach described here (and used also in e.g., [1, 13]) does not appear to be sensitive to maternal state within the timeframe of our collection.

We previously reported that total ChP expression of NKCC1 (*Slc12a2*) in mice increased as development proceeded. Abundance of the activated phosphorylated form of NKCC1 (pNKCC1) is high during the perinatal stage and only decreases after P7, after CSF [K^+^] reaches adult levels [1]. Immunoblotting from rat developing ChP to detect NKCC1/pNKCC1 showed consistent results with mice (Fig. 4E). Total NKCC1 expression in rat ChP increased from embryonic stages to adult, while pNKCC1 was higher at young, perinatal ages (Fig. 4E-G). Together, these analyses indicating conservation between mice and rats of a perinatal developmental decrease in CSF and serum K^+^ concentration and correlation with pNKCC1 levels, support the conclusion that CSF K^+^ clearance by pNKCC1 is a fundamental process during brain development.

## Discussion

The main findings in this follow-up study show that ChP NKCC1 is indeed necessary for CSF K^+^ clearance in perinatal mice and that this regulatory function is conserved across rodent species. We developed and demonstrated the utility of a new NKCC1 conditional knockout mouse line (*flox-Slc12a2*) to assess the effects of NKCC1 loss in the ChP.

The conditional *Slc12a2* knockout allows us to study NKCC1 in a time- and tissue- specific manner. While pharmacological approaches can be used to inhibit NKCC1, including bumetanide and furosemide [19–21], they suffer from low specificity. For example, furosemide nearly equally inhibits NKCC1 and KCC2 [21, 22]. While bumetanide exhibits about 500 x higher affinity for NKCC1 than for KCC2 (K_i_ = 0.1 µM vs. 25–50 µM) and is therefore largely specific to NKCC1 at low doses (i.e., 2–10 µM) [23], bumetanide is lipophilic and its actions are not tissue specific. Genetic approaches can provide greater specificity, although systemic knockout approaches are challenging [24–26], again due to the broad expression of NKCC1 throughout the body (e.g., exocrine glands, inner ear, kidney, neurons, microglia, and ChP). Other conditional NKCC1 knockouts were previously reported in which loxP sites are inserted into introns 7 and 10 of the *Slc12a2* gene [27, 28]. Our model adds to the NKCC1 toolbox with a new *Slc12a2*^*fl/fl*^ mouse strain generated by CRISPR genome editing to target exons 5–6, similar to the manipulation site used in commercially available germline knockout mouse model available at JAX laboratory (exon 6). Because there is no known Cre-line that would exclusively target ChP epithelial cells, we leveraged an AAV-based approach with demonstrated tropism for ChP epithelial cells to achieve ChP NKCC1 knockout. Additionally, because the function and transport direction of NKCC1 during normal CSF secretion and in hydrocephalus has been a recent topic of heated debate [2–11], this ability to specifically knockdown NKCC1 in the ChP alone at any given time may aid the resolution of this complex question. For example, assessing CSF production or ventricular volume after ChP-specific NKCC1 knockdown could help to resolve whether ChP NKCC1 is required for early CSF secretion. Our conditional mouse model could also be applied to study NKCC1 at later developmental stages and/or in disease conditions with reported increase of NKCC1 activation, such as hemorrhagic stroke or infection [2, 29, 30]. Similar to other conditional knockouts, our model can also be applied to knockdown NKCC1 in other cell types, such as immune cells (driven by Cx3cr1-Cre) [28], glial cells (Gfap-Cre), and various subtypes of neurons.

Embryonic NKCC1 knockdown in the ChP impaired CSF K^+^ clearance during neonatal development. Moreover, these effects occurred in the absence of broad developmental changes in the early neocortex indicating that ChP-NKCC1 is not required for cortical neuron or glial generation or gross brain morphology by P4, although later roles may exist as NKCC1 mutations are associated with neurodevelopmental disorder in patients [17, 31]. Despite the higher CSF K^+^ concentrations in NKCC1 conditional knockouts at P4, we found no substantial alteration in cell fate specification. Future studies may reveal whether an extended period of high CSF [K^+^] influences ISF [K^+^] [32] or affects other properties of cortical progenitors, neuronal maturation, or excitability.

Our recapitulation in rats of several key findings in mice is important given the use of rodent models for CSF and hydrocephalus research. Specifically, we found conservation of a perinatal developmental decrease in CSF and serum K^+^ concentration, which correlated with pNKCC1 levels, indicating that CSF K^+^ clearance by pNKCC1 is likely a fundamental process during brain development across species. Such conservation between species lends confidence in using mouse models to study CSF ion and fluid imbalance conditions, such as hydrocephalus in children, and target this developmental window and ChP NKCC1 for intervention. Because the period of CSF K^+^ clearance is quite short in both mice and rats, and may occur at a different time of development in species with different developmental time frames, previous studies of pre- and post-natal CSF [32–35] may not fully capture this process. Previous studies report E19-E21 rat CSF [K^+^] to be closer to adult levels, as measured by placing electrodes within the subarachnoid space of rat embryos stabilized in warm agar. The differences in measuring approaches (total potassium measured by ICP-OES vs. K^+^ concentrations from ion-selective electrodes), CSF sampling approaches (samples CSF from cisterna magna by glass capillaries vs. direct insertion of electrodes within the anatomically restricted subarachnoid space), and potential variability in gestational stages could contribute to the different reporting of [K^+^] values [32, 34]. Similarly, we measured total potassium in serum samples collected by cardiac puncture using ICP-OES, while previous works measured plasma K^+^ concentration using ion-selective electrodes inserted into the transverse sinus. Such differences in methodology (i.e., serum vs. plasma, and ICP-OES vs. electrodes [32, 35]) may contribute to variations in reported absolute values.

Hypoxia during sample collection is one key concern for K^+^ measurements. We found that CSF and serum contained much higher K^+^ when collected from deceased adult animals than those anesthetized by ketamine/xylazine, consistent with hypoxia during sample collection [18, 36]. On the other hand, our side-by-side comparison using mouse embryos from anesthetized dams vs. embryos removed from euthanized dams showed that embryonic CSF was not sensitive to the different collection conditions if embryonic heartbeat was intact, ruling against the possibility of artificially high CSF K^+^ due to hypoxia of the dam. For rats P4 and older, we used ketamine/xylazine for anesthesia which does not interfere with normal breathing. P0-P1 pups are highly sensitive to ketamine/xylazine and were therefore anesthetized by hypothermia and collected within 2–3 min, a timeframe where the pups can rapidly recover by warming. While it is impossible to fully eliminate risk of hypoxia, our data showed the most robust serum K^+^ decrease occurred between E17 to E19, times when samples were collected under the same *in utero* conditions, and only a small fluctuation in serum K^+^ was observed between P1 and older ages. Additionally, samples collected first vs. last within a litter showed no clear trend of increasing K^+^ levels. We conclude that the risk of embryonic and neonatal data being confounded by hypoxia in this study is low.

In summary, we report follow-up studies on ChP NKCC1-mediated developmental CSF K^+^ clearance by ChP-specific NKCC1 knockdown in mice and provide comparative data in rats. Future studies will focus on larger mammals to identify the corresponding time window for each species, including human [35], for translational applications.

## Data Availability

Novel reagents are available from the corresponding author or a designated repository. The datasets used and/or analyzed during the current study are available from the corresponding author on reasonable request.

## List of abbreviations

ChP: choroid plexus
CSF: cerebrospinal fluid
ICP-OES: Inductively Coupled Plasma Optical Emission spectroscopy
NKCC1: Na-K-Cl co-transporter 1
